# Effects of Hydrographic Variability on the Spatial, Seasonal and Diel Diving Patterns of Southern Elephant Seals in the Eastern Weddell Sea

**DOI:** 10.1371/journal.pone.0013816

**Published:** 2010-11-03

**Authors:** Martin Biuw, Ole Anders Nøst, Audun Stien, Qin Zhou, Christian Lydersen, Kit M. Kovacs

**Affiliations:** 1 Norwegian Polar Institute, Polar Environmental Centre, Tromsø, Norway; 2 Norwegian Institute for Nature Research (NINA), Polar Environmental Centre, Tromsø, Norway; National Oceanic and Atmospheric Administration/National Marine Fisheries Service/Southwest Fisheries Science Center, United States of America

## Abstract

Weddell Sea hydrography and circulation is driven by influx of Circumpolar Deep Water (CDW) from the Antarctic Circumpolar Current (ACC) at its eastern margin. Entrainment and upwelling of this high-nutrient, oxygen-depleted water mass within the Weddell Gyre also supports the mesopelagic ecosystem within the gyre and the rich benthic community along the Antarctic shelf. We used Conductivity-Temperature-Depth Satellite Relay Data Loggers (CTD-SRDLs) to examine the importance of hydrographic variability, ice cover and season on the movements and diving behavior of southern elephant seals in the eastern Weddell Sea region during their overwinter feeding trips from Bouvetøya. We developed a model describing diving depth as a function of local time of day to account for diel variation in diving behavior. Seals feeding in pelagic ice-free waters during the summer months displayed clear diel variation, with daytime dives reaching 500-1500 m and night-time targeting of the subsurface temperature and salinity maxima characteristic of CDW around 150–300 meters. This pattern was especially clear in the Weddell Cold and Warm Regimes within the gyre, occurred in the ACC, but was absent at the Dronning Maud Land shelf region where seals fed benthically. Diel variation was almost absent in pelagic feeding areas covered by winter sea ice, where seals targeted deep layers around 500–700 meters. Thus, elephant seals appear to switch between feeding strategies when moving between oceanic regimes or in response to seasonal environmental conditions. While they are on the shelf, they exploit the locally-rich benthic ecosystem, while diel patterns in pelagic waters in summer are probably a response to strong vertical migration patterns within the copepod-based pelagic food web. Behavioral flexibility that permits such switching between different feeding strategies may have important consequences regarding the potential for southern elephant seals to adapt to variability or systematic changes in their environment resulting from climate change.

## Introduction

The Weddell Sea plays an important role in the Southern Ocean circulation system. In addition to being one of the most important regions of Antarctic Bottom Water formation [1] and a globally significant contributor to natural CO2 sequestration [2], the Weddell Sea also supports rich and diverse ecosystems. The influx of Circumpolar Deep Water (CDW) from the Antarctic Circumpolar Current (ACC) at the eastern boundary of the Weddell Sea Gyre plays a central role in all these processes [3]–[4]. Modified CDW, referred to as Warm Deep Water (WDW) within the Weddell Sea, is characterized by distinct subsurface temperature and salinity maxima (*T_max_* and *S_max_*) below the permanent thermocline. The waters associated with these maxima are significantly depleted in oxygen and enriched in nutrients [1], [5]. They also have substantially higher total CO_2_ compared to the source waters to the southeast [2], implying local enrichment due to high biological activity. This local biological activity results from Ekman pumping within the cyclonic Weddell Gyre which causes upwelling of the high-nutrient, low-O_2_ WDW towards the surface, where it stimulates biological primary production and subsequent grazing by zooplankton. This productivity is further increased during ice retreat because of released nutrients and exposure of the water column to light, leading to substantial spring phytoplankton blooms. In addition to the important role this local biological activity can have for the uptake of atmospheric CO2 within the gyre, it is also crucial for supporting the rich and diverse Weddell Sea marine ecosystems. The phytoplankton blooms support a high biomass of grazing zooplankton, and this in turn represents an important food resource for pelagic fish and higher predators. In addition, sinking organic matter provides an important energy pathway between pelagic waters and the benthos; the Weddell Sea benthic community has one of the highest biomasses observed in the Antarctic Ocean [6]. The CDW therefore appears to play a crucial role for physical as well as biological processes within the Weddell Gyre, and water mass distribution and mixing processes are likely to influence the abundance and the horizontal and vertical distribution of organisms in these high-Antarctic marine ecosystems substantially.

The locally elevated primary productivity in pelagic waters in the Weddell Gyre, and the rich benthic communities on the Antarctic continental shelf bordering the gyre to the south, constitute seasonally predictable food resources for resident as well as long-range migrating predators such as seabirds and marine mammals. Penguins and flying seabirds from colonies in Dronning Maud Land forage extensively throughout the eastern Weddell Sea [7]–[8], and a recent study showed that Arctic terns (*Sterna paradisaea*) that breed in Greenland visit this region during the boreal winter [9]. Many Southern Ocean and Antarctic seals also use the region extensively for foraging [10]–[14], and the eastern Weddell Sea has been identified as an important feeding area for large whales [15]. Much of the recent understanding of how marine predators use their environment comes from satellite telemetry and data-logging technologies. This is especially true for long-ranging and deep-diving seabirds and marine mammals that spend most of their life in the open ocean and under the sea surface, effectively making direct observation impossible [7], [16]–[21].

The southern elephant seal (*Mirounga leonina*) is the largest phocid seal and an abundant circumpolar Southern Ocean predator [22]. They are capable of undertaking long-range migrations in search of suitable foraging regions [14], [20], [23]–[26], and represent an important top-trophic component of many Southern Ocean marine ecosystems [27]. While they are not traditionally considered true Antarctic pack-ice seals along with the Weddell (*Leptonychotes wedellii*), crabeater (*Lobodon carcinophagus*), leopard (*Hydrurga leptonyx*) and Ross (*Ommatophoca rossi*) seals, recent tracking studies have shown that a substantial number of elephant seals from key circumpolar populations undertake foraging migrations to the pack ice and the Antarctic continental shelves, often remaining in heavy ice cover throughout the Antarctic winter [14], [23], [28]–[31]. Biuw et al. [23] presented results from the first study that simultaneously tracked southern elephant seals throughout much of their global range, and described a number of general migration and diving strategies, ranging from pelagic foraging trips within the ACC to benthic diving along the coast of east Antarctica and the West Antarctic Peninsula. Southern elephant seals are among the most extreme air-breathing diving predators known, spending over 85% of their time at sea submerged and frequently diving to depths exceeding 1000 m [32]–[33], allowing them to exploit food resources that are inaccessible to most other air-breathing marine predators.

While a number of studies have examined the possible links between southern elephant seal movements and dynamic environmental features such as fronts, eddies and ice distribution [25], [28]–[29], [31], [34], most of these studies have been focused on ocean surface properties assessed via remote sensing data. But, subsurface hydrographic characteristics are known to play an important role in the spatial distribution and variability of primary production and associated trophic dynamics though these characteristics are much more challenging to measure directly, in-situ and in real time; few studies to date have been able to assess their effects on deep diving species such as elephant seals. However, the recent development of satellite-linked instruments capable of recording and relaying high-quality hydrographic data directly from the animals themselves [35]–[37] have proven extremely valuable in this regard, while also providing a valuable complementary technique for physical oceanographic studies [12], [30], [36]–[42]. Recent studies using this novel instrumentation have demonstrated that the diving behavior and foraging performance of southern elephant seals can be strongly influenced by vertical variation in temperature and salinity [23], [31], [37], [43]. Biuw et al. [23] showed that water characteristics targeted by elephant seals varied substantially between populations and general oceanic regions in a circumpolar context. Furthermore, Bailleul et al. [43] showed that elephant seals from the Kerguelen Islands specifically targeted colder waters in regions where foraging was successful, passing quickly through waters of similar depth in other regions where foraging was less successful.

Biuw et al. [23] provided a general description of variation in southern elephant seal diving behavior in relation to time of day, with deeper diving occurring during daylight hours and shallower diving at night. Diel vertical migration (DVM) has been described for organisms from a wide range of taxa (see [44], and references therein). In Antarctic waters, studies of copepods [45]–[52], krill [53]–[55] and to a lesser degree also mesopelagic fish [56] have described such patterns. Diel variation in diving behavior of many Southern Ocean and Antarctic marine top predators, including elephant seals [13], [32], [57]–[67], are generally assumed to reflect the DVMs of their potential prey or underlying trophic components [60], [62], [64], [68]–[70], but there have been no detailed accounts of the links between specific hydrographic features, zooplankton and fish DVMs and the diel variation in diving of air-breathing vertebrates, and as yet no quantitative studies of diel variations in diving and their seasonal and regional patterns of occurrence.

While our understanding of the wide-ranging and circumpolar distribution of southern elephant seals has increased in recent years, some geographic gaps remain, most notably in the Weddell Sea. While some few elephant seals tracked from various colonies have entered this area, these movements appear to have been exceptions rather than common occurrences. Bouvetøya (54°25′ S 3°21′ E), situated on the Atlantic-Indian Ridge is home to a small elephant seal colony that is more likely to utilize regions within the Weddell Sea compared to more distant colonies at for instance South Georgia or the Prince Edward Islands. However, no studies of the at-sea movements of Bouvetøya elephant seals had been carried out prior this study, conducted during the most recent Norwegian Antarctic Research Expedition (NARE) to the island in 2007–2008.

In this paper we describe the general movements of southern elephant seals from the small, remote colony at Bouvetøya. We also examine to what degree the diving behavior of Southern elephant seals in the southeast Atlantic sector of the Southern Ocean and the Weddell Sea is influenced by *in-situ* hydrographic properties, addressing in particular the importance of the Circumpolar Deep Water to the diving behavior of foraging elephant seals in this region. To provide a more quantitative assessment of the seasonal and spatial patterns of occurrence of diel dive variation (DDV), we also develop a model of diving behavior as a function of time of day. We examine 1) whether the presence of DDV is related to the general oceanic regime within which a seal is operating; 2) if there is any evidence that seals target specific water masses (such as the CDW), and if such a preference exists, whether it is related to the presence of DDV; and 3) if the occurrence of DDV is influenced by environmental variability other than water mass preference or general oceanic region. Specifically, we explore whether there are any effects of season or ice extent/concentration on the occurrence of DDV? We discuss these findings in the context of currently available information about seasonal and vertical patterns of occurrence of Weddell Sea zooplankton and fish, and discuss how predator diving patterns can provide some insights into these patterns which may help direct future ecosystem studies in this region.

## Materials and Methods

Table 1 provides a summary of acronyms used in the text.

**Table 1:** Acronyms used in the text to denote technical terms, ocean regimes, water masses, hydrographic features and model parameters.

Acronym	Explanation
CTD	Conductivity-Temperature-Depth
SRDL	Satellite Relay Data Logger
ACC	Antarctic Circumpolar Current
CDW	Circumpolar Deep Water
WDW	Warm Deep Water
ACCR	Antarctic Circumpolar Current Regime
WCR	Weddell Cold Regime
WWR	Weddell Warm Regime
SR	Dronning Maud Land Shelf Regime
ASF	Antarctic Slope Front
FPL	Four-parameter logistic function
*T_max_*	Depth of subsurface temperature maximum
*S_max_*	Depth of subsurface salinity maximum
*D_night_*	Night-time asymptotic dive depth
*D_day_*	Daytime asymptotic dive depth
*t_mid_*	Local time corresponding to inflection point of four-parameter logistic function
*λ*	Scaling parameter proportional to the maximum rate of change in depth at *t_mid_*
DDV	Diel dive variation

### Animal handling and instrument deployment

Twenty Conductivity-Temperature-Depth Satellite Relay Data Loggers (CTD-SRDLs, Sea Mammal Research Unit, St Andrews, UK) were deployed on southern elephant seals at Bouvetøya in the Atlantic sector of the Southern Ocean (Fig. 1A) in February 2008, during the Norwegian Antarctic Research Expedition (NARE) to the island. Animals were captured at the end of the annual molt and were chemically immobilized and handled according to previously described methods [71]–[73]. For details on instrument specifications see Boehme et al. [35], and for general information regarding onboard data compression and transmission strategies see Fedak et al.[74]. All animal handling was conducted in accordance with the Regulation of Animal Experimentation of the Norwegian Animal Research Authority under permit number 2007/1932.

**Figure 1:**
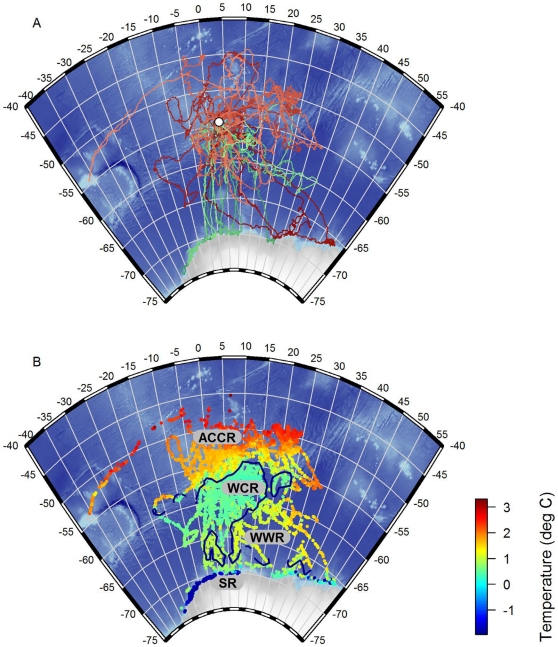
Bouvetøya elephant seal tracks and seal-derived subsurface temperature maximum. A) Tracks of 19 southern elephant seals instrumented at Bouvetøya (grey diamond) covering the period February -November 2008. Green tones  =  males, red tones  =  females. B) Positions of 8749 CTD profiles obtained throughout the tracking period. The color represents the temperature at the subsurface maximum (*T_max_*). The dark blue contour line represents the 0.8°C isotherm used to delineate between the Antarctic Circumpolar Current Regime (ACCR), Weddell Cold Regime (WCR) and Weddell Warm Regime (WWR). The delineation between the WWR and the Dronning Maud Land Shelf regime (SR) was based on the location of the Antarctic Slope front (ASF).

### Environmental covariates

**Figure 2:**
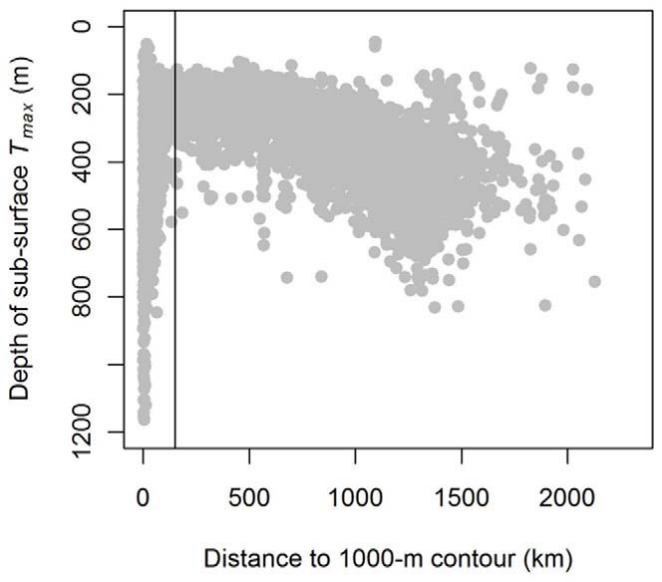
Depth of subsurface temperature maximum (*T_max_*) vs. distance from Dronning Maud Land Shelf 1000-m contour. The vertical line represents a distance of 150 km which was used to delineate between the Weddell Warm Regime (WWR) and the Dronning Maud Land Shelf Regime (SR). This distance was chosen based on the location of the Antarctic Slope Front (ASF) which is associated with the sharp deepening of the (*T_max_*).

To examine the extent to which habitat use and diving behavior of southern elephant seals in the eastern Weddell Sea region can be described as a function of both static and dynamic environmental characteristics, we defined a series of environmental covariates based on hydrographic data collected by the seals, bottom topography from the ETOPO1 1 arc-minute Global Relief Model [75] and ice cover data obtained from the SSMI PSI 12.5 km gridded sea ice product [76]. Following the general large-scale circulation patterns [3]–[4] and broad ecological regions described for the Weddell Sea [52], we divided the study area (defined as the region of the Eastern Weddell Sea visited by the seals from Bouvetøya) into four broad oceanic regimes (Fig. 1B): the Antarctic Circumpolar Current Regime (ACCR); the Weddell Cold Regime (WCR); the Weddell Warm Regime (WWR); and the Dronning Maud Land Shelf Regime (SR). We used the temperature at *T_max_* for each profile obtained from the seals to delineate between the ACCR, WCR and WWR. The Weddell Front is situated at the southern edge of the mid-Atlantic ridge, and marks the transition from ACC waters to WCR waters [4]. This transition is characterized by a sharp horizontal gradient in temperature at *T_max_* from 1.5°C to 0.5°C [77]. Smedsrud [78] has shown that the WDW has warmed from ∼0.6°C to r 0.9°C between 1975 and 2000. We used a relatively conservative value of 0.8°C as our delineation between the ACCR and WCR in the north and also between the WCR and WWR in the southsimilar to Spiridonov et al. [52] who used 0.75°C for this southern delineation. To distinguish the WWR from the SR to the south, we determined the horizontal location of the Antarctic Slope Front (ASF) relative to the 1000-m bathymetric contour by identifying the rapid deepening of the *T_max_* from the CTD profiles obtained from the seals (Fig. 2).

### Analytical and statistical approach

**Figure 3:**
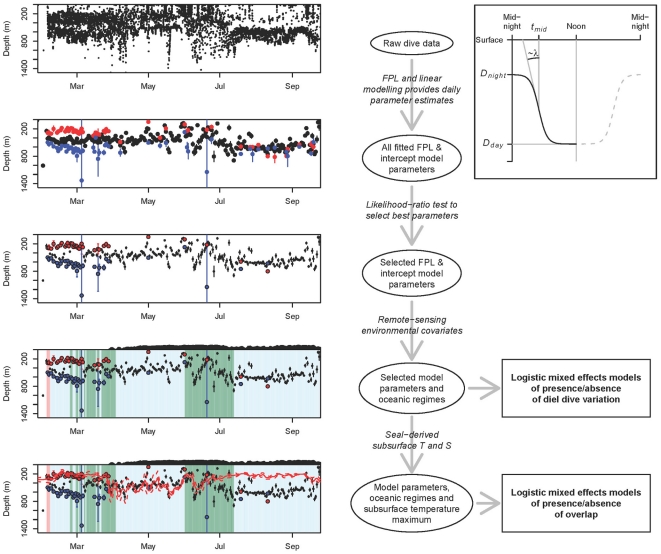
Schematic flow-chart of the analytical approach. The strip-charts in the left column represent time vs depth (cf. Fig. 6). The top figure shows the raw data for maximum depth reached during individual dives throughout the sampling record of one seal and subsequent strip-charts illustrate the output of applying the successive procedures indicated in italics in the middle column. The inset (top right-hand) illustrates the parameters of the four-parameter logistic model of diving depth as a function of time of day, as defined in Eq. 1. The *λ* symbol indicates that the maximum slope of the curve is determined by the *λ* parameter. The two squares (bottom right-hand) represent the two series of final logistic mixed effects models testing for relationships between dive patterns and environmental covariates.

All data analyses were done using R version 2.10.1 [79]. Our analyses proceeded in a step-wise fashion, which is outlined in Figure 3. To obtain representative daily parameters of depth preference we first tested for the presence or absence of any diurnal variation in dive depth (DDV) by comparing a sigmoidal vs an intercept-only linear model of dive depth as a function of time of day. The sigmoidal model was based on a four-parameter logistic function (FPL): 
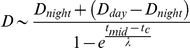
where *D_night_* and *D_day_* denote the expected night-and daytime dive depths respectively, *t_c_* is the observed time (see below), *t_mid_* is the local time corresponding to the inflection point of the curve (i.e. where the rate of change in *D* is greatest), and *λ* represents a scaling parameter setting the maximum rate of change in *D* (see Fig. 3 (inset) for a graphical representation of these parameters). Rather than using a double logistic function to cover the 24 hr cycle, we transformed local time *t* to reflect its temporal distance from local midnight: 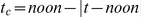



The main advantage of this transformation is that it provides more data for the fitting of the nonlinear model. However, it assumes that *D_night_* is the same at both extremes of the time range (i.e. at 00:00 and 24:00), and that the inflection point and rate of inflection are also the same at dawn and dusk. While these assumptions are not met in all circumstances (for instance due to sunset and sunrise not always being symmetrically distributed around local noon), any deviations from such dawn/dusk symmetry are unlikely to introduce any major biases at the scale of our analysis. To obtain reliable estimates of daily *D_night_*, *D_day_*, *t_mid_* and *λ* parameter values, the FPL fitting was done in two steps. Initial parameter estimates were obtained using a robust nonlinear regression model by M-estimators, using iterated reweighted least squares. This approach is implemented in the nlrob function available in the robustbase package for R [80]. If this model converged, indicating the presence of a diel pattern for a specific seal on a given day, these initial parameter estimates were used to fit a final model using the nls function (in the standard stats package for R). The reason for this two-stage approach is that parameter estimates from the the nls function are much more reliable, but that convergence of this function can be heavily dependent on providing appropriate parameter starting values. The FPL model was fitted for each seal day separately along with the ‘flat’ linear model that included a simple intercept parameter which provided an estimated average diving depth for a given day, irrespective of the time of day. The presence of a DDV pattern was evaluated by comparing the two models using a likelihood-ratio test, where support for the FPL model was assessed against a χ^2^ distribution with the relevant degrees of freedom, using p = 0.05 as the significance threshold.

Once the presence/absence of DDV had been determined, we fitted two series of logistic mixed effects models (Fig. 3). The first series tested for the presence/absence of DDV in relation to the spatio-temporal environmental covariates described above, while the second series examined the degree to which seals targeted the *T_max_*. In this second series of models, the fitted night-time asymptote of the FPL or the fitted intercept of the linear model was used, depending on the degree of support for a diel pattern on a given day. The second series also tested if the degree to which seals targeted the *T_max_* was influenced by any of the environmental covariates. In the first series of models, the response variable was therefore the presence or absence of a diel pattern (i.e. a significant or non-significant result in the likelihood-ratio test described above). In the second series of models, the binomial response variable represented the presence or absence of an overlap between the mean ±1 sd of the location of the *T_max_* and the mean ±1 se of the parameter estimate (i.e. *D_night_* parameter if a diel pattern was observed and the linear intercept parameter if no diel pattern was observed). We did not test for overlap between *D_day_* and *T_max_* since visual inspection of the data indicated that such an overlap was either extremely infrequent or absent entirely for all individuals. Individual seal was included as random effect in these models, to allow for the lack of independence between data points for individual seals. All logistic mixed effects models were fitted using the lmer function for fitting linear or generalized linear mixed models (in the lme4 package for R [81]).

## Results

**Figure 4:**
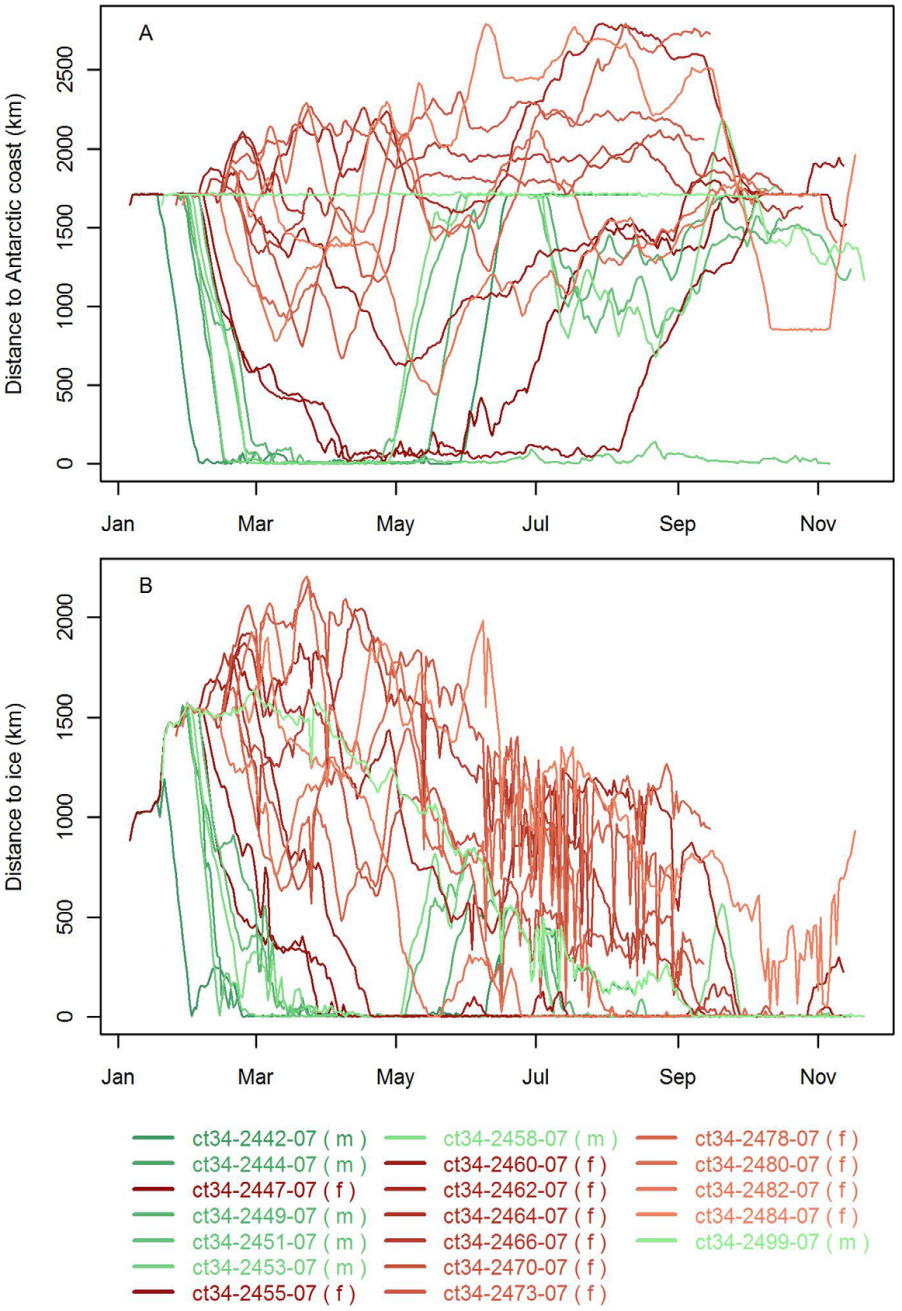
Distance to A) the Antarctic coastline and B) edge of the pack ice. Time-traces of males and females throughout the tracking period are illustrated in green and red tones respectively.

One of the twenty instruments stopped transmitting within the first week, as the seal carrying the instrument was buried in an ice fall/land slide before the seal had left the island. The remaining 19 instruments were deployed on 7 subadult males and 12 were adult females. Instruments provided data for an average of 248 days (range = 47–306 days). After departing from Bouvetøya seals followed one of two general migration patterns (Fig. 1A and Fig. 4A). Six subadult males travelled south along relatively direct trajectories toward the Dronning Maud Land Shelf Regime (SR). Two adult females also travelled directly southwards until they reached the frontal region between the Weddell Cold Regime (WCR) and Weddell Warm Regime (WWR), where they remained for several weeks before they too continued to the shelf. The remaining 11 seals (10 females and 1 male) stayed further north, mostly in the pelagic waters of the ACCR or WCR. Only a few of these individuals ventured southward into the WWR, and they were in these areas for only short periods. Nevertheless, even seals that remained in pelagic waters to the north interacted with sea ice as the ice extended northwards gradually throughout the winter months (Fig. 4B). One male remained within 50 km of Bouvetøya for the first eight months following instrumentation, until early October when it travelled southeast until the instrument stopped transmitting on November 21. Eleven seals returned to Bouvetøya, either for a midwinter haulout (4 males) or for the breeding season (7 females). One adult female that spent the winter feeding to the east and north of Bouvetøya travelled to South Georgia for the breeding season, where she hauled out in Hound Bay on the northeast coast of the island for ∼3 weeks before she travelled northeast, backtracking her inward migration to South Georgia, until her instrument stopped transmitting on November 18.

**Figure 5:**
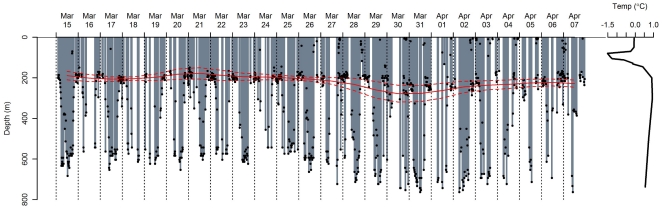
Diving strip-chart for adult female southern elephant seal from Bouvetøya. Stripchart represents a 40-day period during the early stage of the post-moult trip. Black filled circles represent the maximum depths of individual dives. Vertical broken lines represent local midnight. The solid and broken red lines represent the time-weighted means and standard deviations of the depth of the sub-surface water temperature maximum, corresponding to Circumpolar Deep Water (CDW) or Weddell Sea Deep Water (WSDW) depending on the geographic location. The temperature profile on the right corresponds to data collected during the deepest dive of this 40-day period (registered 2^nd^ Apr).

**Figure 6:**
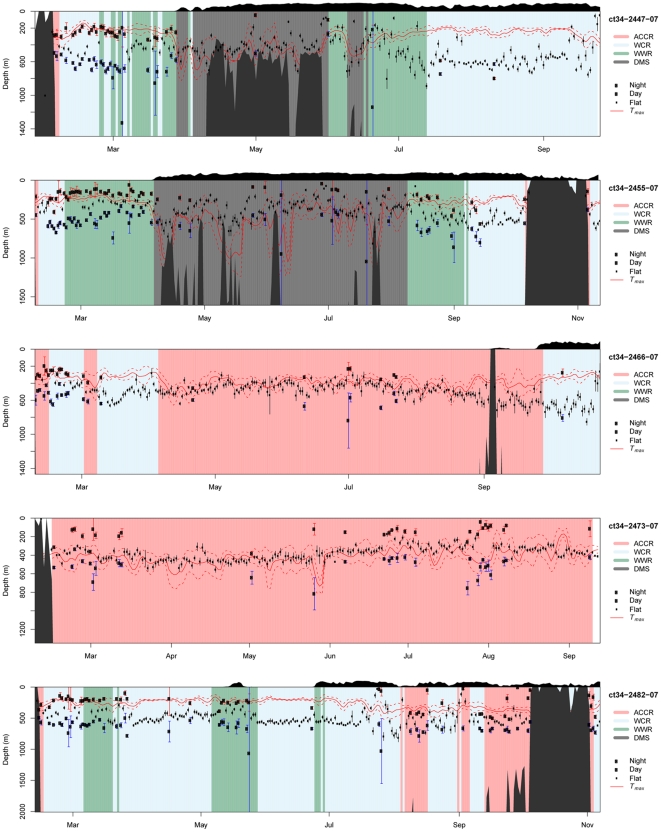
Example time-depth strip-charts from five female southern elephant seals. Strip charts show females migrating between Bouvetøya and: Dronning Maud Land (top two charts), the Background colors indicate which ocean regime a seal is in on a given day. Red and blue filled circles represent the fitted shallow and deep asymptotes of the four-parameter logistic for days when there was strong support for the existence of a diel pattern, while the smaller black circles represent the fitted intercept in the linear intercept-only models for days when support for a diel pattern was weak. Vertical error bars associated with these points represent standard errors of the fitted parameters. The solid and broken red lines represent the average and standard deviation of the depth of the subsurface temperature maximum (*T_max_*) of the Warm Deep Water. The black region at the top of each strip-chart represents relative ice concentration while black regions within the strip-charts represent the seafloor.

Figure 5 shows the diving performance of one seal while it was in the WCR, illustrating the typical pattern of diel dive variation (DDV) performed by seals within the Weddell Gyre during late summer and autumn. Daytime dives reached the Weddell Sea Deep Water (WSDW) 500700 meters, well below the *T_max_*, while night-time dives clearly targeted the *T_max_* associated with the WDW. This pattern was never observed when seals were over the shelf region, and was mostly limited to the initial 2 months after seals departed from Bouvetøya. This was supported by the likelihood-ratio tests, which indicated better fits for the four-parameter logistic model compared to the intercept model during this early period, while the ‘flat’ linear intercept model generally provided better fits later in the sampling period. Figure 6 shows the diving behavior of five seals, illustrating the presence/absence of DDV in relation to hydrography and topography. The top two panels present data from two females that travelled south to the frontal region between the WCR and WWR before proceeding to the SR along the central and eastern Dronning Maud Land coast. The diel dive pattern and the night-time targeting of the WDW can be seen clearly from February until April, at which time the seals reached the SR and commenced benthic diving. When seals left the shelf again during mid-late winter, they resumed pelagic feeding in the now ice-covered WCR and WWR. At this time DDV was almost absent, and seals dove through the WDW, targeting the WSDW at 500–700 m during both day and night. The next two panels show data from two females that remained further north within the WCR and particularly the ACCR. These two examples clearly show targeting of CDW, irrespective of presence or absence of DDV. The bottom panel shows data from a female that moved between the WCR, WWR and ACCR, and shows clear DDV and targeting of the CDW/WDW during the early period, and focused diving to deep layers beyond the *T_max_* during the winter months.

**Figure 7:**
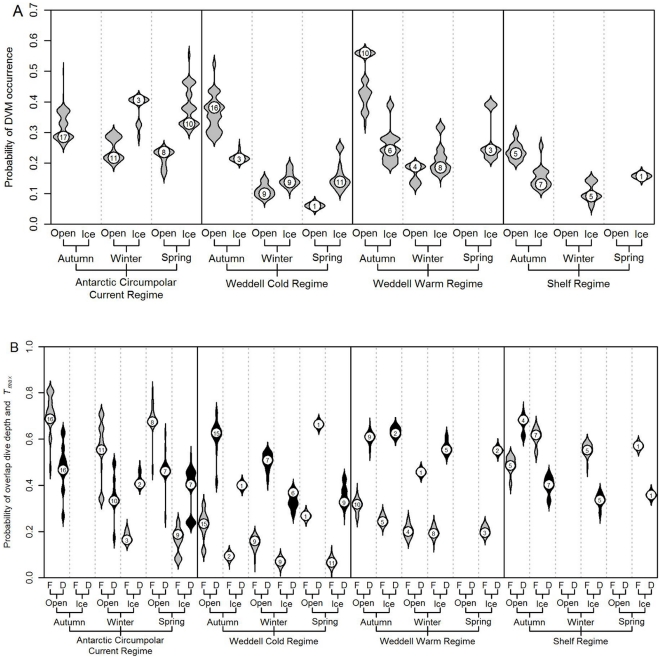
Predicted probabilities of occurrence of diel dive variation and dive depth and *T_max_* overlap. A) occurrence of diel dive variation (DDV) and B) overlap between dive depth and depth of the subsurface temperature maximum (*T_max_*) across oceanic regimes (ACCR, WCR, WWR and SR), seasons (Autumn, Winter, Spring) and ice conditions (Open water or Ice) encountered by southern elephant seals from Bouvetøya during their winter post-moult migrations. The relationships in A and B are based on the top scoring among the logistic mixed effects models presented in Tables 1 and 3 respectively. Shapes represent relative probability densities and numbers within the shapes are the number of individual seals which occupied each given combination of conditions. In Fig. 7B, ‘F’ and ‘D’ refers to ‘Flat’ and ‘Diel’ respectively, i.e. the absence or presence of DDV.

The models estimating the probability of observing DDV as a function of temporal and environmental covariates are compared in Table 2. Among these models, the occurrence of DDV was best described by a model including regime, season and ice concentration along with the interaction terms for season:regime and season:ice concentration. According to this model the occurrence of DDVs is predicted to be lower during winter and spring compared to late summer and autumn, especially in the WCR and WWR (Fig. 7A). Within these two regimes the presence of sea ice in autumn further reduces the probability of DDV. Within the SR, the occurrence of DDV is consistently low, especially from winter onwards and in the presence of sea ice. Table 3 presents the comparison of models estimating the probability of overlap between the vertical position of *T_max_* and the depths targeted by seals. The best supported model among the candidates included fixed terms for regime, season, ice and presence of DDV, along with the 2-way interactions ice:season, ice:regime, ice:DDV, regime:DDV and the 3-way interaction regime:ice:DDV. This model predicts that within the ACCR, the probability of overlap between *T_max_* and dive depth is generally high in open water regardless of season, while the presence of sea ice in winter and spring reduces this probability of overlap. It also suggests that while in the ACCR, overlap in ice free regions is more associated with flat diel dive patterns, while the presence of sea ice appears to favour overlap on days with DDV. Within the WCR and WWR, the probability of overlap is particularly high in autumn during days when seals perform DDV. While the probability is predicted to be generally lower in winter and spring, the presence of DDV appears to increase the probability to some degree. This implies that when seals perform DDV within these two central Weddell regimes, they clearly target the WDW during dives performed at night. Within the SR, the model predicts the probability of overlap to be consistently relatively high. This simply reflects the fact that while on the shelf, seals tend to dive to the bottom, which is also where *T_max_* occurs as a result of intrusions of CDW or modified CDW along the bottom.

**Table 2:** Candidate logistic mixed effects models of the occurrence of diel dive variation of southern elephant seals instrumented on Bouvetøya in February 2008.

Model	*log*ℒ	AIC	ΔAIC	Rel ℒ	AICw
ice + (regime × season) + (season × ice)	−1844.8	3721.6	0.00	1.000	0.605
ice + (regime × season) + (regime × ice) + (season × ice)	−1842.4	3722.8	1.23	0.542	0.328
ice + (regime × season) + (regime × ice)	−1846.2	3726.4	4.83	0.089	0.054
season + (regime ×ice)	−1854.1	3730.3	8.68	0.013	0.008
ice + (regime × season)	−1852.2	3732.5	10.86	0.004	0.003
(ice × season) + (ice ×regime)	−1853.3	3732.6	11.02	0.004	0.002
Regime × season	−1857.5	3741.0	19.37	0.000	0.000
regime + (ice × season)	−1860.6	3741.3	19.65	0.000	0.000
regime + season + ice	−1864.0	3743.9	22.31	0.000	0.000
regime + season	−1869.7	3753.5	31.87	0.000	0.000
regime × ice	−1874.2	3766.4	44.82	0.000	0.000
regime + ice	−1892.2	3796.4	74.76	0.000	0.000
regime	−1910.3	3830.6	109.02	0.000	0.000
season × ice	−2178.3	4370.6	648.94	0.000	0.000
season + ice	−2185.8	4381.7	660.06	0.000	0.000
ice	−2203.8	4413.5	691.90	0.000	0.000
season	−2205.8	4419.5	697.92	0.000	0.000

*log* ℒ: Model log-likelihood; ΔAIC: Difference in AIC relative to model with lowest AIC value; Rel ℒ: Relative likelihood; AICw: AIC weight.

**Table 3:** Candidate logistic mixed effects models estimating the occurrence of overlap between the mean 1 standard deviation of the location of the Tmax and the mean 1 standard error of the parameter estimate pattern was or was not observed).

Model	*log* ℒ	AIC	ΔAIC	Rel ℒ	AICw
(regime * season * diel) + (diel * ice) + (regime * ice) + ice	2046.8	4153.6	0.00	1.000	0.710
(regime * season * diel) + (diel * ice) + (regime * ice) + (season * ice) + ice	2046.0	4156.0	2.41	0.300	0.213
(regime * season * diel) + (regime * ice) + ice	2050.3	4158.7	5.10	0.078	0.055
(regime * season * diel) + (regime * ice) + (season * ice)	2049.7	4161.5	7.89	0.019	0.014
(regime * season * diel) + (season * ice) + ice	2053.3	4162.7	9.10	0.011	0.008
(regime * season * diel) + (diel * ice) + ice	2060.6	4175.3	21.67	0.000	0.000
(regime * season * diel) + ice	2063.5	4179.0	25.39	0.000	0.000
(regime * season * diel)	2077.9	4205.9	52.26	0.000	0.000

(i.e. Dnight or the linear intercept parameter respectively, if a diel pattern was or was not observed).

*log* ℒ: Model log-likelihood; ΔAIC: Difference in AIC relative to model with lowest AIC value; Rel ℒ: Relative likelihood; AICw: AIC weight.

## Discussion

This study shows that the eastern Weddell Sea is an important post-molt and post-breeding foraging area for southern elephant seals from the nearby colony at Bouvetøya, and confirms that this large Southern Ocean marine predator is an important component of ecosystems within the high latitude Antarctic gyre systems such as the Weddell Gyre, where heavy pack ice is a prominent feature. The study also demonstrates clearly how the behavior of this extreme diver is influenced strongly by local hydrography, ice cover, season and time of day.

### General movement patterns and habitat selection

Although southern elephant seals are not considered to be among the pack ice seals, it is now clear that many elephant seals are associated closely with the Antarctic shelf, the ice edge and even the interior of the seasonal pack ice, and that the strength of such associations depends in part on age and sex [14], [23], [28]–[29], [31], this study. Sexual segregation in movement patterns and habitat selection by southern elephant seals was first described by McConnell et al. [20] for animals from South Georgia; subsequently, these phenomena have also been reported for other populations [24], [28], [31], [82]–[83]. Bailleul et al. [28], [31] found that among young elephant seals from the Kerguelen Islands, males tended to spend more time feeding along the coast and shelf of East Antarctica while females more often remained in the frontal regions within the ACC. The small number of females that did visit the Antarctic shelf regions in their study tended to follow the ice edge as this expanded northwards during late autumn and winter, while males tended to remain on the shelf in heavy pack ice, sometimes throughout the entire Antarctic winter [28], [31]. These authors suggested that such segregation may be explained by lifetime fitness costs for failing to return to sub-Antarctic breeding colonies in a given season being higher for females than for males, thereby making it more important for females to avoid the risk of getting trapped by the expanding pack ice by moving northwards as it expands. Because of the small sample size in our study we did not specifically address sexual segregation. While some general sexual differences were apparent, our data were characterized more by large variations in foraging strategies, between as well as within individuals. In our study, 6 out of 7 males travelled to the Dronning Maud Land Shelf for some time, but only one male definitely remained there throughout the entire winter. Four males returned to Bouvetøya for a midwinter haulout (May/June), before they embarked on a second feeding trip along or slightly south of the edge of the pack ice, ∼1500 km north of the Antarctic coastline, in regions also utilized by many of the female seals.

The differences between the results reported by us and those of Bailleul et al. [28], [31] could be due to the age of the study animals. While female elephant seals start reproducing in their 3^rd^ or 4^th^ year of life, males become reproductively active much later, probably at an age of at least 7–8 years [22]. The juvenile males studied by Bailleul et al. [28], [31] were therefore too young to be reproductively active, whereas the subadult males in our study were approaching the age when they might start attending the breeding colonies as challengers. The tendency of these subadult males to move north with the ice and switch from Antarctic shelf feeding to feeding pelagically in the pack ice within reach of open water, suggests that males approaching reproductive age tend to avoid the risk of being trapped by pack ice along the Antarctic coast, in the same way adult females do. The females in our study generally conformed to the pattern described by Bailleul et al. [28], [31]. Most of the females (10) remained in open water or in association with the edge of the pack ice. However, the two females that travelled to the Antarctic coastline remained well within the pack ice until they returned to Bouvetøya just prior to the breeding season in late September/early October.

### Vertical movements and ecosystem links

Due to our relatively incomplete understanding of ecosystem structure and trophic linkages in high-Antarctic systems, and our fragmentary knowledge about the diet of southern elephant seals, we can only speculate about the nature of the intermediate factors that link seal distribution and diving to hydrographic conditions at this time. However, the fact that we find clear and consistent links between specific hydrographic features, ice distribution and seal diving behavior, in combination with some knowledge of lower trophic animals in the system allows us to make some preliminary suggestions regarding hot-spots and ecological linkages within this region. Unlike many other high-productivity regions in the Southern Ocean, krill does not constitute a key component of the Weddell Sea ecosystems. Instead, copepods are extremely abundant and probably form the zooplankton base of pelagic ecosystems within the Weddell Gyre [49]. Calanoid copepods in the Weddell Sea display pronounced seasonal vertical migrations, spending the summer in warm surface waters and generally overwintering in the Warm Deep Water below 500 m (see [49], and references therein). Spiridonov et al. [52] described a bimodal vertical distribution of calanoid copepods in pelagic waters of the Weddell Sea at the onset of winter. In addition to high abundances of mostly younger and smaller specimens in surface waters, a second peak of larger and older age classes was found around or below the *T_max_* in pelagic regions north of the shelf break. This bimodal pattern was particularly clear within the Weddell Gyre and close to the Weddell Front, where the core copepod abundance was found in the Lower Circumpolar Deep Water at depths of 400–700 m. This might explain our finding that elephant seals feeding in the WCR and WWR during winter target the ∼500–700 m water layers, well below the *T_max_*. It is likely that the high abundances of copepods represent profitable winter food resources for mesopelagic fish such as myctophids [84]–[86], which are likely consumed directly by elephant seals, or which might constitute an intermediate link to other elephant seal prey species such as cephalopods and larger mesopelagic fish [87]. In a study based on stable isotopes in the blood of female elephant seals from Kerguelen Islands, Cherel et al. [88] suggested that myctophids may play a much more important role in their diet than previously assumed. They suggested further that elephant seals might occupy a unique trophic niche among air-breathing vertebrates, feeding on mesopelagic fish in deep waters that are inaccessible to most other air-breathing divers. Flores et al. [89] found high abundances of myctophids in the offshore community of the Weddell Sea, and suggested that the vertical migrations of these species (especially *Electrona antarcticus*) may represent a major energy transfer mechanism between surface waters and deeper layers in the Lazarev Sea food-web. It is possible therefore that myctophids also represent an important prey resource for elephant seals in the pelagic Weddell Sea ecosystems.

Mesopelagic Antarctic fish species other than myctophids (e.g. Antarctic deepsea smelt, *Bathylagus antarcticus*; veiled anglemouth, *Cyclothone microdon*; Antarctic jonasfish, *Notolepis Coatsi*) are also known to occur in high densities in water layers around 500–1000 [56], [90]. High abundances of cephalopods, particularly glacier squid (*Psychroteuthis glacialis*) and *Alluroteuthis antarcticus* which are quite abundant in the central and southern Weddell Sea, can also be found at similar depths (Piatkowski, pers. comm.). Glacier squid have been identified as an important prey species for elephant seals that breed at King George Island [91], and also for emperor penguins (*Aptenodytes forsteri*) in the eastern Weddell Sea and elsewhere [65], [92]. While emperor penguins are able to dive to depths of 300–500 m [64]–[65], the extreme diving capability of elephant seals likely gives may give them a substantial advantage in accessing this prey. If deep water masses are indeed important over-wintering layers for zooplankton, mesopelagic fish or squid, this would constitute a very rich and predictable resource for elephant seals, especially during the critical winter foraging period prior to the energetically demanding spring breeding period.

In addition to the clear winter-targeting by elephant seals of specific water masses and vertical layers, our study also provides a thorough analysis of the factors affecting the presence or absence of diel variation in the depths layers they target. DDVs are tightly coupled to hydrographic features, specifically the subsurface *T_max_* of the CDW, but season and other more general environmental conditions also play a role. While DDVs were very common in pelagic waters during the summer and early autumn, they were virtually absent during winter and in the presence of sea ice, and seals instead targeted deeper layers irrespective of the time of day. This may reflect seasonal changes in zooplankton vertical distribution or changes in their tendency to perform DVMs. These movements are presumably also mirrored in the vertical distribution of their mesopelagic fish predators [85].

Transitions between daytime and night-time dive depths were closely timed with sunset and sunrise, suggesting that seals target prey that show light-driven vertical migrations during periods of clear day-night cycles. Cisewski et al [46] described zooplankton DVMs in the Lazarev Sea from late summer to spring, showing clear night-time ascents into the surface layers at night and descents into the ∼200–300 m layer during daytime. While the diel variation in diving by elephant seals show the same timing, the depth layers were not the same. At night, when elephant seals began to target the ∼200–400 m layers associated with the *T_max_* corresponding to the core of the CDW, the bulk of the zooplankton biomass already appeared to have ascended into shallower surface layers. However, the clear night-time targeting of the CDW by elephant seals suggests that high biomasses of mesopelagic fish or cephalopods still occupy this layer at night, presumably supported by sufficient densities of their zooplankton prey which therefore must also remain in these mid-water layers rather than ascending into the surface waters. Some species of calanoid copepods in Antarctic waters are known to remain at high densities in mid-water layers [49] where they may constitute a food resource for mesopelagic fish and cephalopods irrespective of season and time of day. Several studies have also described size and age related variations in the vertical distribution of zooplankton and mesopelagic fish [49], [52], [90], with larger species generally being more abundant in deeper layers. For instance, Antarctic silverfish (*Pleuragramma antarcticum*), which is by far the most abundant pelagic fish species in Antarctic shelf waters, and occupies a wide range of water depths from the surface to ∼900 m [93]. While mainly a shelf species with adults spawning in nearshore waters, Antarctic silverfish larvae are believed to migrate into offshore pelagic waters where they may remain in the Weddell Gyre for several years before returning to the shelf [94]–[95]. In terms of vertical distribution, they show clear age/size stratification, with small larvae in the surface layers and the largest specimens found around 500–700 m depth [90]. Antarctic silverfish are considered a keystone species in the Antarctic seasonal pack ice zone [96], and have been recorded in the diet of several Antarctic marine top predators, including elephant seals at colonies in the South Shetland Islands [97].

### The shelf system

The four male and two female elephant seals that fed on the shelf along Dronning Maud Land fed benthically, diving to depths of 400–500 m. There is no specific information regarding what the seals might be eating at this time, but glacier squid are frequently encountered in benthopelagic trawls along the shelf slope in the eastern Weddell Sea and are the main cephalopod prey of elephant seals from the South Shetland Islands [91]. Fish species such as Antarctic silverfish and Antarctic toothfish (*Dissostichus mawsoni*) might also be important food for the seals in this near-shore shelf system [98]–[99].

### Flexibility in movements and feeding habits

Southern elephant seals display a wide range of feeding strategies and movement patterns, both between and within individuals. While high degrees of individual site fidelity has been observed in many studies, and explained in terms of potential fitness consequences [100], individuals also display substantial variations in habitat use and movement patterns between and even within feeding trips. Such flexibility might also be important in terms of foraging performance and might have indirect effects on reproductive success, especially for organisms inhabiting temporally dynamic environments. For instance, flexibility in foraging ranges and diet have been suggested as an explanation for stable reproductive success of northern gannets over periods of fluctuating reproductive success of many other seabird species in the region [101]. Eelephant sealsmight have even more flexibility and ability to adjust to spatial variations and temporal changes in prey abundance and distribution because they can remain at sea for such long periods. Switches between feeding strategies, such as those observed for the 4 males feeding on the shelf in autumn and early winter and moving north into pelagic waters in late winter and spring, are likely a reflection of such flexibility, which may in fact be a more important and particular characteristic of elephant seal foraging behavior than individual site fidelity. The ability to switch between feeding strategies might allow elephant seals to adapt with relative ease to environmental variability, perhaps even rapid climate change.

### Future perspectives

It is clear that southern elephant seals play an important role as top consumers in mesopelagic and benthic shelf ecosystems, from the northern reaches of the Southern Ocean to high-latitude Antarctic waters, such as those in the eastern Weddell Sea. It is therefore crucial that they are taken into account in efforts to model and understand trophic linkages in Southern Ocean and high-Antarctic ecosystems. Traditionally, krill has been the primary focus of Antarctic ecosystems models and management, but there is a growing concern that this focus has unduly masked the existing variability and ecosystem diversity in the Southern Ocean. While krill are known to be present in the eastern Weddell Sea, the much more abundant copepods probably play a more fundamental role in the ecosystems of the region [49]. Mesopelagic fish (e.g. myctophids) and cephalopods are likely linked with copepod distribution, abundance and life history, and important top predators such as elephant seals, may be dependent on these linkages. Our understanding of these ecosystems is still too fragmentary to quantify their trophic dynamics but integrated process studies of subsurface ocean physics, biogeochemistry, primary production, zooplankton and mid-trophic dynamics and top predator distribution and behavior should be explored in order to advance ourunderstanding and management of the living resources in these remote but highly productive ocean regions.
